# CoryneRegNet 7, the reference database and analysis platform for corynebacterial gene regulatory networks

**DOI:** 10.1038/s41597-020-0484-9

**Published:** 2020-05-11

**Authors:** Mariana Teixeira Dornelles Parise, Doglas Parise, Rodrigo Bentes Kato, Josch Konstantin Pauling, Andreas Tauch, Vasco Ariston de Carvalho Azevedo, Jan Baumbach

**Affiliations:** 1 Institute of Biological Sciences, Universidade Federal de Minas Gerais, Belo Horizonte, Minas Gerais Brazil; 2 Chair of Experimental Bioinformatics, TUM School of Life Sciences, Technical University of Munich, Munich, Germany; 3 LipiTUM, Chair of Experimental Bioinformatics, TUM School of Life Sciences, Technical University of Munich, Munich, Germany; 4 Center for Biotechnology (CeBiTec), Bielefeld University, Bielefeld, Germany

**Keywords:** Gene regulatory networks, Genetic databases, Bacteriology

## Abstract

We present the newest version of CoryneRegNet, the reference database for corynebacterial regulatory interactions, available at www.exbio.wzw.tum.de/coryneregnet/. The exponential growth of next-generation sequencing data in recent years has allowed a better understanding of bacterial molecular mechanisms. Transcriptional regulation is one of the most important mechanisms for bacterial adaptation and survival. These mechanisms may be understood via an organism’s network of regulatory interactions. Although the *Corynebacterium* genus is important in medical, veterinary and biotechnological research, little is known concerning the transcriptional regulation of these bacteria. Here, we unravel transcriptional regulatory networks (TRNs) for 224 corynebacterial strains by utilizing genome-scale transfer of TRNs from four model organisms and assigning statistical significance values to all predicted regulations. As a result, the number of corynebacterial strains with TRNs increased twenty times and the back-end and front-end were reimplemented to support new features as well as future database growth. CoryneRegNet 7 is the largest TRN database for the *Corynebacterium* genus and aids in elucidating transcriptional mechanisms enabling adaptation, survival and infection.

## Introduction

Next-generation sequencing (NGS) has unraveled the genomic sequence of a multitude of bacterial genomes^1^. Despite the amount of information, these data do not fully explain how organisms orchestrate their survival on a molecular level. To understand the mechanisms that coordinate an organism’s adaptation to environmental changes, it is crucial to understand how a cell maintains transcription^2,3^. The main players in the transcriptional regulation of bacterial organisms are transcription factors (TFs). These regulatory proteins recognize transcription factor binding sites (TFBSs) in the upstream region of the respective target genes (TGs), stimulating or repressing their expression^4–6^. Experimental studies such as RNA-Seq^7^, microarray^8^, ChIP-chip and ChIP-seq^9^ have been applied in order to reveal regulatory interactions in a cell. Nevertheless, performing these experiments for all bacterial strains would be labor-intensive and, thus, financially infeasible^4,10^. As a result, these experimental data are not available for every member of a bacterial genus.

**Fig. 1:**
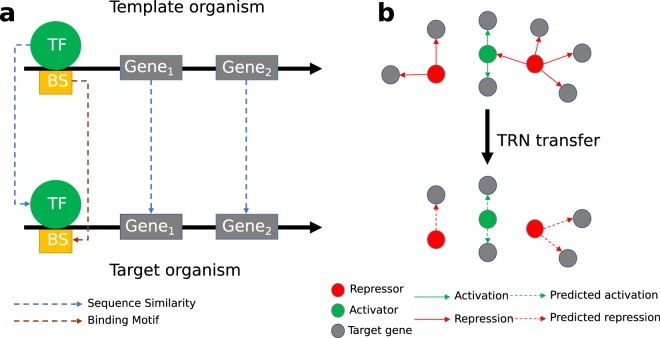
Overview of the computational reconstruction of TRNs. (**a**) General concept of regulatory conservation. (**b**) TRN transfer scheme: The TRN of a model organism (top) and the predicted TRN of the target organism including all transferred regulations (bottom). In the networks, nodes represent the genes and arrows represent the regulatory interactions.

To alleviate this lack of data, genome-scale transfer of TRNs has been applied providing insights into the regulatory mechanisms of bacterial organisms^10,11^. In this context, a model organism is an organism with the most complete and experimentally validated TRN which can be utilized to predict regulatory interactions in other organisms, called target organisms, with incomplete or less validated TRNs. TRNs are constructed as directed graphs where the nodes represent TFs and their TGs while the criterion to create an edge from a TF to a TG is the regulatory interaction between them^6,12,13^. Edge labels may then indicate the corresponding TFBSs and/or the type of the regulatory interaction. A reliable method to transfer TRNs from a model organism to taxonomically related target organisms is to consider a regulatory interaction to be conserved between two organisms when the TF, the TG and the TFBS are all conserved^4^. Figure 1a illustrates a conserved regulatory interaction between two organisms. An example of a TRN transferred from a model organism to a target organism is represented in Fig. 1b. More extensive explanations on genome-scale TRN transfer methods can be found in Baumbach *et al*.^11^ and Kiliç *et al*.^14^.

Both experimentally and computationally reconstructed TRNs are publicly available in databases such as RegulonDB^15^ for *Escherichia coli*, EHECRegNet^16^ for human pathogenic *Escherichia coli*, TB Portal^17^ and MTB Network Portal^18^ for *Mycobacterium tuberculosis*, DBTBS^19^ and Subtiwiki^20^ for *Bacillus subtilis*, and CoryneRegNet^21^ for the *Corynebacterium* genus. RegulonDB^15^ focuses on detailed and manually curated transcriptional regulation data retrieved from literature for *E. coli*. MTB Network Portal^18^ and Subtiwiki^20^ provide literature-mined transcriptional regulation data on *M. tuberculosis* and *B. subtilis*. Abasy Atlas^22^ is an online collection of regulatory data covering 42 bacteria retrieved from both literature and other online databases. However, there is no resource focusing on corynebacterial gene regulatory networks, and no database that stores predicted TRNs based on evolutionary conservation across a whole collection of model and target organisms. CoryneRegNet has served as the reference database of the genus *Corynebacterium* since 2006^23^. This genus includes organisms with medical, veterinary and biotechnological relevance^24–27^. While the National Center for Biotechnology Information (NCBI) database contains more than 60 corynebacterial species with fully sequenced and annotated genomes, there are TRNs of only eight of these species available in online databases.

The previous version of CoryneRegNet^21^ was released in 2012 presenting predicted (transferred) TRNs for eleven corynebacterial strains. The steady increase in corynebacterial genomic sequences contained in public databases allows us to unravel further transcriptional regulatory interactions. In the seventh version of CoryneRegNet, we now present 82,268 regulatory interactions, an increase of more than eleven times compared to the sixth version, as well as 228 TRNs, increasing the number of corynebacterial strains with known TRNs by twenty times. It contains up-to-date regulatory information about the model organisms *C. glutamicum* ATCC 13032, *E. coli* K-12, *M. tuberculosis* H37Rv and *B. subtilis* 168, and predicted TRNs of 224 target organisms of the *Corynebacterium* genus. Furthermore, we present an increase of more than seven times the number of corynebacterial species with TRNs available in public databases representing a great improvement for the bacterial gene regulatory network research community.

## Results

In this section we present the results of the re-implemented back- and front-end, the updated database content and the predicted TRNs of all fully sequenced and annotated corynebacterial genomes.

### Updated database content

In CoryneRegNet 7 we updated the database content by adding new model and target organisms. As in previous versions, TRNs are categorized as either experimentally validated or computationally predicted. The former contains up-to-date TRNs of *C. glutamicum* ATCC 13032, *E. coli* K-12, *M. tuberculosis* H37Rv and *B. subtilis* 168, the latter contains predicted TRNs of a total of 224 corynebacterial strains. A full list of these strains as well as more details regarding the experimental and predicted databases are presented in Supplementary Table S1. The resulting number of predicted TFs, regulated genes, regulations, binding motifs and profile Hidden Markov Models (HMMs) are presented in Table 1 together with the evolution of the database content throughout previous CoryneRegNet versions.

**Table 1:** Content of experimental (e) and predicted (p) databases in this version and previous versions of CoryneRegNet.

Version	Organisms	TFs	Reg. genes	Regulations	BMs	PWMs	profile HMMs	Publication
1.0	1	53	331	430	192	23	—	Baumbach *et al*.^23^
2.0	4	64	499	607	274	29	—	Baumbach *et al*.^41^
3.0	5	213	1632	2912	1522	130	—	Baumbach *et al*.^53^
4.0	7	213	1632	2912	1522	130	—	Baumbach *et al*.^54^
5.0e	11	245	1986	3712	1759	144	—	—
5.0p	11	350	2888	4928	2553	249	—	—
6.0e	12	245	1986	3712	1759	144	—	Pauling *et. al*.^21^
6.0p	12	482	3946	6352	3429	381	—	Pauling *et. al*.^21^
7.0e	4*	539	3921	8162	4974	—	446	This
7.0p	228	9590	57747	82268	56870	—	9497	This

TFs, transcription factors; Reg. genes, regulated genes; BMs, binding motifs; PWMs position weight matrices; profile HMMs, profile hidden Markov models. Adapted from Pauling *et al*.^21^. *Please note that in previous versions of CoryneRegNet, all organisms were considered experimentally validated if their **genomes** had been experimentally verified through sequencing assays. However, to be more accurate, we changed this definition in this version. We now only consider an organism as experimentally validated if its experimentally verified **TRN** is available. This change of definition led to a decrease in the number of actual model organisms in the experimental view. In previous terms this number would have been 228.

### Novel back- and front-end

**Fig. 2:**
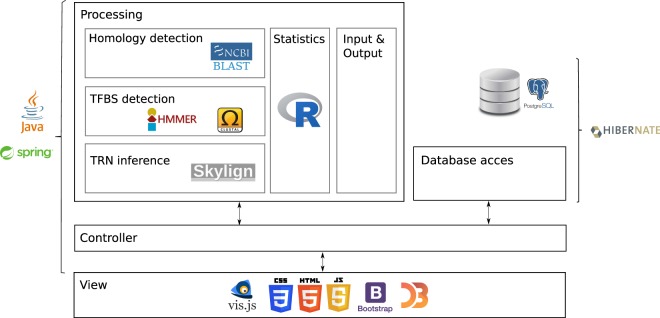
Schematic of the CoryneRegNet 7 architecture. The processing layer handles the TRN transfer and data parsing. The database access layer manages any query, update, insertion or deletion in the database. The view is responsible for user interaction through the browser. The controller conducts all the communication and data handling among the other three layers.

In this work, we re-implemented CoryneRegNet’s back- and front-end, allowing the user to browse the database via a modern and easy-to-use web-interface. The new architecture (Fig. 2) is inspired by the Model-View-Controller (MVC) architectural pattern^28,29^. This modular structure allows components to be modified or replaced, facilitating maintenance and future updates.

### CoryneRegNet web-interface

**Fig. 3:**
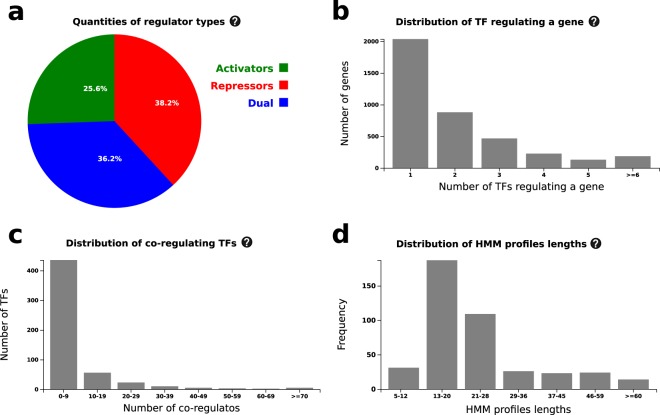
Overview of main statistics present in CoryneRegNet. In (**a**) the pie chart presents the quantities of regulator types in percentages, (**b**) represents the distribution of the numbers of TFs regulating a gene, (**c**) presents the distribution of co-regulating TFs, and (**d**) demonstrates the distribution of HMM profiles lengths.

The CoryneRegNet 7 website contains TRNs of 224 corynebacterial target genomes and 4 model organisms. Information about quantities of regulator types, distribution of TFs, distribution of co-regulating TFs and distribution of HMM profile lengths are shown in the statistics page. Those are shown for each database (predicted and experimental) as well as for each organism. Figure 3 represents the statistics page for the experimental database.

**Fig. 4:**
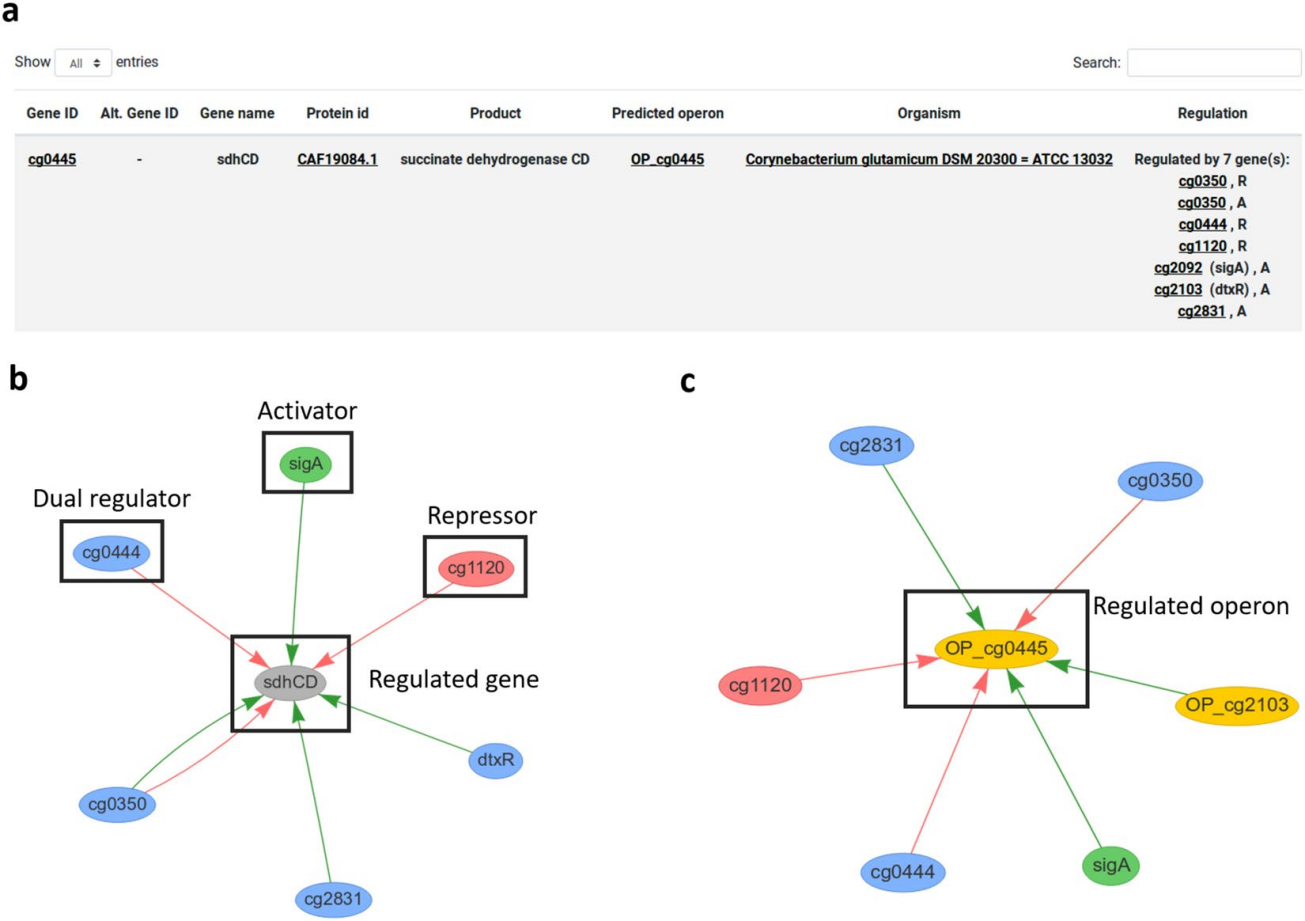
Example of a RI. (**a**) TG (cg0445) and its regulators in table format. (**b**,**c**) present the same TG as in (**a**), but in a graph format with gene-centered layout (**b**) and an operon-centered layout (**c**).

Through the web-interface, the user is able to browse the TRNs in both table and network format. In table format, the list of regulatory interactions (RIs) provides source, target and operon information (Fig. 4a). The network visualization comes with two different layout options: a gene-centered layout and an operon-centered layout, see Fig. 4b,c, respectively. Both visualizations give access to gene information by clicking on the gene and/or operon of interest. Each network may also be downloaded in.sif file format. Additionally, the user can visualize networks of genes or operons of interest by using the network visualization in the gene information pop-up.

**Fig. 5:**
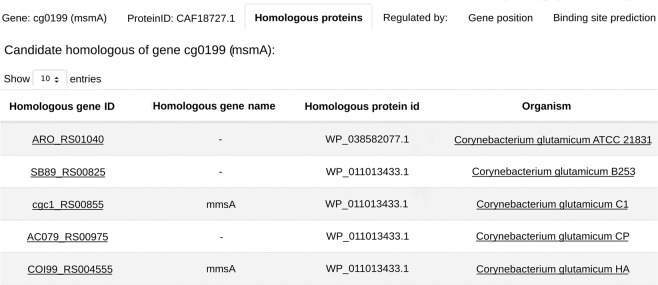
Detailed gene information page. Here, the “Homologous proteins” tab lists homologs of the cg0199 protein in various other organisms.

Furthermore, we offer a detailed gene information page which shows gene identifiers linked to NCBI, nucleotide and protein sequences, homologous genes, and regulatory information. This page also allows the user to make additional motif searches in the database. A view of this page showing the putative homologous genes of cg0199 is presented in Fig. 5. The user is provided with information of all genes that are predicted to be homologous to the gene of interest that are present in CoryneRegNet 7.

**Fig. 6:**
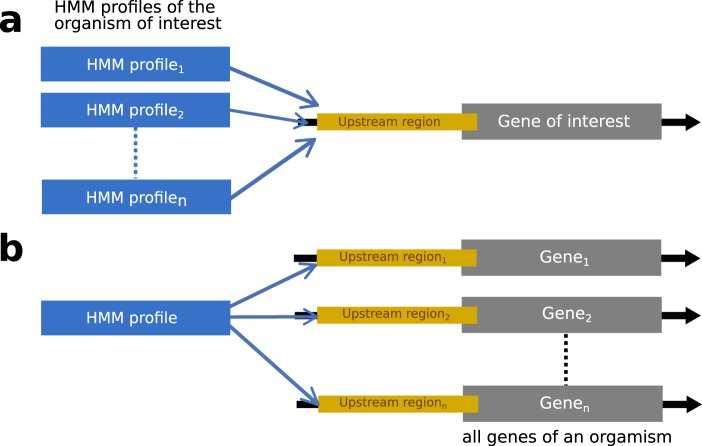
Kinds of motif search provided in CoryneRegNet 7. (**a**) HMM profiles of one organism being used to identify potential binding sites in the upstream region of a gene of interest. (**b**) HMM profile of interest being used to identify potential binding sites in all genes of an organism.

At this point, CoryneRegNet 7 offers the biggest collection of profile HMMs publicly available for the *Corynebacterium* genus. See the methodology section for an explanation of how those were generated. These profiles and their logos are available for download in the gene information page of genes encoding transcription factors. In addition, the user can utilize the profile HMMs stored in the database to search the upstream region of genes present in CoryneRegNet 7. Two kinds of motif searches are provided: (i) the upstream region of the gene of interest can be searched with HMM profiles of an organism of interest (Fig. 6a) and (ii) the HMM profile of the TF of interest can then be used to identify potential binding sites in the upstream regions of all genes of an organism in the database (Fig. 6b).

Finally, the website provides a comprehensive help page with theoretical and practical explanations of the website content, methodology and navigation, including a broad collection of published literature concerning Corynebacterial transcriptional regulation. This help page can be accessed at www.exbio.wzw.tum.de/coryneregnet/docs&help.htm.

## Discussion

In CoryneRegNet version 7, we entirely redesigned the back- and front-end to support the updated database content, new functional features as well as future database growth. As we did in the other versions, we present TRNs for all fully sequenced and annotated corynebacterial genomes available in NCBI (June 2019). Consequently, CoryneRegNet 7 currently offers the biggest knowledge base available regarding TRNs of corynebacterial organisms. Along with our newly-designed web interface, we include an operon network layout and the option to download network views from each organisms network visualization as a file (.sif). This allows users to modify and enrich the network locally and create personalized visualizations based on their own research using third-party software.

We also significantly improved our transfer pipeline in two ways. First, we replaced the use of Position Weight Matrices (PWMs) with profile HMMs in our motif conservation analysis. Profile HMMs enable the modelling of insertions and deletions, greatly improving the detection of remote homologous and model nucleotide dependency as well as length variations in the model’s binding sites^30–32^. TFBSs are considered to have low evolutionary conservation between species^11^ and this strategy provides more robustness and flexibility when predicting them^33^. It is an advantage considering that mutations in these sites are expected to occur from one species to another. Second, we added the calculation of p-values, i.e. the likelihood of observing this conservation by chance, for each regulatory interaction which provides important information in interpreting the results.

Even though great progress has been made in the TRN field, there are still a few limitations concerning bacterial TRNs. The transfer of TRNs has been hindered by the limited availability of experimentally validated data on bacterial TRNs which are available for only a few model organisms such as *E. coli*, *B. subitilis* and *C. glutamicum*^4,14^. TRN transfer from one organism to another largely depends on known regulatory interactions in the model organism as well as genome similarity between model and target organism^4,11,13,14^. Thus, the more experimental TRN data is available for a greater diversity of bacterial species, the higher the quality of any predicted TRNs. Furthermore, the ability of detecting regulatory interactions acquired by horizontal gene transfer (HGT) becomes relevant^4,11,14^ considering regulatory interactions related to life-style may not be identified by using only one model organism. Using several model organisms allows us to identify these regulations. Previous studies point out that (i) known virulence determinants in Enterohemorrhagic *E. coli* are located on mobile genetic elements, which are generally acquired in HGT events^16^ and (ii) in *E. coli* neighboring regulators were co-transferred with their TGs in HGT^34^. Methodologies that support the use of more model organisms together with more high quality experimentally validated TRNs will result in more complete TRNs that will consider lifestyle-related regulatory interactions (e.g. pathogenic or non-pathogenic, free-living or host-associated). A first step in this direction was presented in this study, since we transferred regulatory interactions between organisms of different phyla and presented the joint p-value allowing the researcher to evaluate the degree of conservation of each predicted regulatory interaction.

## Methods

### Database content update

Genomic data of the 228 organisms used in this work were retrieved from the NCBI database^35^ in June 2019 (for more details see Supplementary Table S1). TRN data of the model organisms were retrieved from RegulonDB^15^ for *E. coli* K-12, Minch *et al*.^36^ for *M. tuberculosis* H37Rv, DBTBS^19^ for *B. subtilis*. *C. glutamicum* ATCC 13032 data from CoryneRegNet 6^21^ were updated with new data from Freyre-González and Tauch (2017)^37^.

**Fig. 7:**
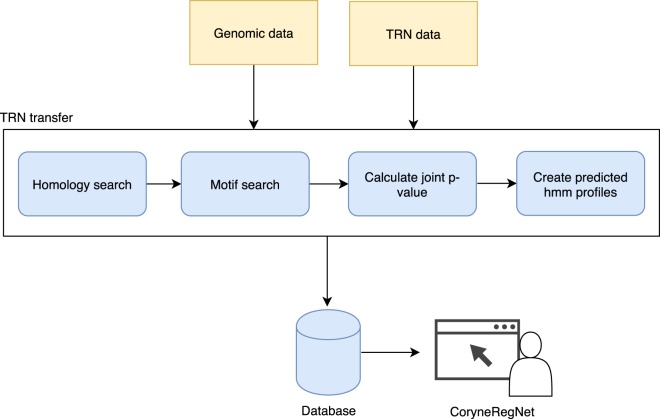
Schematic overview of the TRN transfer pipeline.

In order to predict TRNs for the 224 corynebacterial strains, we extended the transfer methodology described by Baumbach and collaborators^4^. First, TF binding profiles were generated for every TF of the model organisms. For this purpose, binding sites of each TF were collected, aligned with Clustal Omega^38^ and binding profiles were generated using HMM-build from the HMMER package^39^. Second, we performed all-vs-all protein BLAST^40^ search and selected the best bidirectional BLAST hits (BBHs) using a cutoff of 10^−10^ to predict homologous proteins. The upstream regions (−560, +20) of all genes and operons in the analysis were identified. Third, the upstream regions of all homologous TGs in the target organisms were scanned using HMMER^30^ to predict conserved TFBSs. Figure 1a illustrates this approach. The HMM profiles of the conserved TFs were applied to the upstream regions of the potentially regulated TGs by using HMMER’s default parameters, which corresponds to a p-value of ~10^−5^ ^41^. Genes with an intergenic distance of less than 50 base pairs were considered to be part of the same operon and predicted regulatory interactions to the first gene were extended to the operon^4^. The role of a predicted regulatory interaction is inherited from the model regulatory interaction used in the transfer. Finally, profile HMMs were generated for predicted TFs as described above for model TFs. The interaction p-value was obtained by applying Tippet’s method^42^. The R package Metap^43^ was used to calculate the joint p-value of the p-values obtained in the homology and motif searches. These steps are summarized in Fig. 7.

### Implementation of CoryneRegNet 7

The CoryneRegNet7 back-end was developed using Java and the database was implemented using the PostgreSQL^44^ management system for relational databases. Hibernate^45^ was used for object-relational mapping between PostgreSQL and Java. The Spring framework^46^ was used to implement a Model-View-Controller (MVC) architectural pattern^28,29^. The new front-end was developed using HTML5^47^, CSS3^48^, Bootstrap^49^ and JavaScript. We used vis.js^50^ to implement the network visualization and graphs in the statistics page were drawn using D3^51^. The website was tested and runs on Google Chrome and Mozilla Firefox. Furthermore, the screen resolution was optimized for tablet (1024 × 768), laptop (1920 × 1080) and desktop (2560 × 1440) resolutions.

## Supplementary information

Supplementary Table S1

## Data Availability

All data generated in this work is provided to the research community free of charge as comma separated values (.csv format) via the figshare repository^52^ and in CoryneRegNet’s download section (http://www.exbio.wzw.tum.de/coryneregnet/processToDownalod.htm).

## Code availability

CoryneRegNet7 code is available on GitHub: https://github.com/baumbachlab/CoryneRegNet7.
