# Supplementary material for: CoryneRegNet 7, the reference database and analysis platform for corynebacterial gene regulatory networks
Source: Sci Data. 2020 May 11;7:142. doi: 10.1038/s41597-020-0484-9 (PMC7214426; doi:10.1038/s41597-020-0484-9)
Supplement: Supplementary file 1 — Supplementary Table S1 [file 41597_2020_484_MOESM1_ESM.pdf]

Supplementary Table S1 - Organisms

| Organism | Type | Present in CoryneRegNet 6 | Acession number |
|---|---|---|---|
| *Corynebacterium glutamicum* DSM 20300 = ATCC 13032 | model | x | BX927147.1 |
| *Bacillus subtilis* 168 | model | | NC_000964.3 |
| *Mycobacterium tuberculosis* H37Rv | model | | AL123456.3 |
| *Escherichia coli* K-12 | model | x | NC_000913.3 |
| *Corynebacterium diphtheriae* NCTC 13129 | target | x | NC_002935.2 |
| *Corynebacterium diphtheriae* HC03 | target | | NC_016787.1 |
| *Corynebacterium stationis* 622=DSM 20302 | target | | NZ_CP009251.1 |
| *Corynebacterium pseudotuberculosis* 46 | target | | NZ_CP015190.1 |
| *Corynebacterium epidermidicanis* DSM 45586 | target | | NZ_CP011541.1 |
| *Corynebacterium crudilactis* JZ16 | target | | NZ_CP015622.1 |
| *Corynebacterium sp.* ATCC 6931 | target | | NZ_CP008913.1 |
| *Corynebacterium pseudotuberculosis* CAP3W | target | | NZ_CP026500.1 |
| *Corynebacterium striatum* 216 | target | | NZ_CP024932.1 |
| *Corynebacterium diphtheriae* PW8 | target | | NC_016789.1 |
| *Corynebacterium pseudotuberculosis* 38 | target | | NZ_CP015187.1 |
| *Corynebacterium pseudotuberculosis* 38MAT | target | | NZ_CP036457.1 |
| *Corynebacterium pseudotuberculosis* MEX25 | target | | NZ_CP013697.1 |
| *Corynebacterium efficiens* YS-314 | target | x | NC_004369.1 |
| *Corynebacterium pseudotuberculosis* ATCC 19410 | target | | NZ_CP021251.1 |
| *Corynebacterium diphtheriae* BH8 | target | | NC_016800.1 |
| *Corynebacterium pseudotuberculosis* CAPNAT1 | target | | NZ_CP035716.1 |
| *Corynebacterium glutamicum* TQ2223 | target | | NZ_CP020658.1 |
| *Corynebacterium glutamicum* ATCC 21831 | target | | NZ_CP007722.1 |
| *Corynebacterium casei* LMG S-19264 | target | | NZ_CP004350.1 |
| *Corynebacterium pseudotuberculosis* 39 | target | | NZ_CP015188.1 |
| *Corynebacterium kroppenstedtii* DSM 44385 | target | x | NC_012704.1 |
| *Corynebacterium pseudotuberculosis* 42/02-A | target | | NC_017306.1 |
| *Corynebacterium glutamicum* MB001 | target | | NC_022040.1 |
| *Corynebacterium glutamicum* ATCC 13869 | target | | NZ_CP016335.1 |
| *Corynebacterium pseudotuberculosis* MB278 | target | | NZ_CP023395.1 |
| *Corynebacterium segmentosum* NCTC934 | target | | NZ_LR134408.1 |
| *Corynebacterium pseudotuberculosis* PA08 A19 | target | | NZ_CP024602.1 |
| *Corynebacterium ulcerans* 131002 | target | | NZ_CP011095.1 |
| *Corynebacterium diphtheriae* VA01 | target | | NC_016790.1 |
| *Corynebacterium pseudotuberculosis* NCTC4681 | target | | NZ_LR590479.1 |
| *Corynebacterium ammoniagenes* KCCM 40472 | target | | NZ_CP019705.1 |
| *Corynebacterium sp.* 2183 | target | | NZ_CP026947.1 |
| *Corynebacterium pseudotuberculosis* 48 | target | | NZ_CP015191.1 |
| *Corynebacterium pseudotuberculosis* Cap1R | target | | NZ_CP036258.1 |
| *Corynebacterium diphtheriae* NCTC3529 | target | | NZ_LR134538.1 |

| | | | |
|---|---|---|---|
| *Corynebacterium pseudotuberculosis* 34 | target | | NZ_CP015192.1 |
| *Corynebacterium camporealensis* CIP 105508 | target | | NZ_CP027001.1 |
| *Corynebacterium pseudotuberculosis* Cap8W | target | | NZ_CP036257.1 |
| *Corynebacterium pseudotuberculosis* 43 | target | | NZ_CP015189.1 |
| *Corynebacterium kutscheri* NCTC949 | target | | NZ_LR134377.1 |
| *Corynebacterium kutscheri* NCTC11138 | target | | NZ_LR134381.1 |
| *Corynebacterium callunae* DSM 20147 | target | | NC_020506.1 |
| *Corynebacterium glutamicum* B253 | target | | NZ_CP010451.1 |
| *Corynebacterium pseudotuberculosis* 1002B | target | | NZ_CP012837.1 |
| *Corynebacterium glutamicum* N24 | target | | NZ_AP017369.1 |
| *Corynebacterium imitans* DSM 44264 | target | | NZ_CP009211.1 |
| *Corynebacterium pseudotuberculosis* MEX31 | target | | NZ_CP017292.1 |
| *Corynebacterium pseudotuberculosis* PAT10 | target | | NC_017305.1 |
| *Corynebacterium glutamicum* C1 | target | | NZ_CP017995.1 |
| *Corynebacterium lactis* RW2-5 | target | | NZ_CP006841.1 |
| *Corynebacterium pseudotuberculosis* 316 | target | | NC_016932.1 |
| *Corynebacterium pseudotuberculosis* N1 | target | | NZ_CP013146.1 |
| *Corynebacterium glutamicum* AJ1511 | target | | NZ_AP017557.1 |
| *Corynebacterium glutamicum* USDA-ARS-USMARC-56828 | target | | NZ_CP013991.1 |
| *Corynebacterium pseudotuberculosis* CAPMI05 | target | | NZ_CP035718.1 |
| *Corynebacterium ulcerans* 0102 | target | | NC_018101.1 |
| *Corynebacterium pseudotuberculosis* 48252 | target | | NZ_CP008922.1 |
| *Corynebacterium ulcerans* NCTC7910 | target | | NZ_LT906443.1 |
| *Corynebacterium pseudotuberculosis* MB66 | target | | NZ_CP013263.1 |
| *Corynebacterium pseudotuberculosis* PO222/4-1 | target | | NZ_CP013698.1 |
| *Corynebacterium diphtheriae* FDAARGOS_197 | target | | NZ_CP020410.2 |
| *Corynebacterium pseudotuberculosis* MIC6 | target | | NZ_CP019769.1 |
| *Corynebacterium resistens* DSM 45100 | target | | NC_015673.1 |
| *Corynebacterium diphtheriae* ISS 3319 | target | | NZ_CP025209.1 |
| *Corynebacterium urealyticum* DSM 7109 | target | x | NC_010545.1 |
| *Corynebacterium pseudotuberculosis* I37 | target | | NZ_CP017384.1 |
| *Corynebacterium striatum* KC-Na-01 | target | | NZ_CP021252.1 |
| *Corynebacterium pseudotuberculosis* Ft_2193/67 | target | | NZ_CP008924.1 |
| *Corynebacterium diphtheriae* HC02 | target | | NC_016802.1 |
| *Corynebacterium diphtheriae* TH1526 | target | | NZ_CP038504.1 |
| *Corynebacterium pseudotuberculosis* 32 | target | | NZ_CP015183.1 |
| *Corynebacterium pseudotuberculosis* CAPMI03 | target | | NZ_CP035717.1 |
| *Corynebacterium pseudotuberculosis* 258 | target | | NC_017945.2 |
| *Corynebacterium sphenisci* DSM 44792 | target | | NZ_CP009248.1 |
| *Corynebacterium pseudotuberculosis* Cp13 | target | | NZ_CP014998.1 |
| *Corynebacterium ulcerans* NCTC7908 | target | | NZ_LS483400.1 |
| *Corynebacterium renale* NCTC11140 | target | | NZ_LS483404.1 |
| *Corynebacterium falsenii* BL 8171 | target | | NZ_CP007156.1 |

| | | | |
|---|---|---|---|
| *Corynebacterium argentoratense* DSM 44202 | target | | NC_022198.1 |
| *Corynebacterium glutamicum* YI | target | | NZ_CP014984.1 |
| *Corynebacterium pseudotuberculosis* Cap1W | target | | NZ_CP034411.1 |
| *Corynebacterium kutscheri* NCTC3655 | target | | NZ_LR134407.1 |
| *Corynebacterium glutamicum* WM001 | target | | NZ_CP022394.1 |
| *Corynebacterium pseudotuberculosis* PA07 | target | | NZ_CP024457.1 |
| *Corynebacterium glutamicum* AR1 | target | | NZ_CP007724.1 |
| *Corynebacterium singulare* IBS B52218 | target | | NZ_CP010827.1 |
| *[Brevibacterium] flavum* ZL-1 | target | | NZ_CP004046.1 |
| *Corynebacterium pseudotuberculosis* 36 | target | | NZ_CP015186.1 |
| *Corynebacterium urealyticum* DSM 7111 | target | | NC_020230.1 |
| *Corynebacterium stationis* LMG 21670 | target | | NZ_CP019963.1 |
| *Corynebacterium sp.* NML98-0116 | target | | NZ_CP017639.1 |
| *Corynebacterium pseudotuberculosis* 04MAT | target | | NZ_CP036469.1 |
| *Corynebacterium stationis* ATCC 6872 | target | | NZ_CP014279.1 |
| *Corynebacterium pseudotuberculosis* Cp162 | target | | NC_018019.2 |
| *Corynebacterium ulcerans* FRC11 | target | | NZ_CP009622.1 |
| *Corynebacterium pseudotuberculosis* Cap4W | target | | NZ_CP039867.1 |
| *Corynebacterium pseudotuberculosis* E56 | target | | NZ_CP013699.1 |
| *Corynebacterium glutamicum* ATCC 14067 | target | | NZ_CP022614.1 |
| *Corynebacterium ulcerans* 131001 | target | | NZ_CP010818.1 |
| *Corynebacterium pseudotuberculosis* PO269-5 | target | | NZ_CP012695.1 |
| *Corynebacterium glutamicum* R | target | x | NC_009342.1 |
| *Corynebacterium pseudotuberculosis* 1002 | target | x | NC_017300.1 |
| *Corynebacterium pseudotuberculosis* E19 | target | | NZ_CP012136.1 |
| *Corynebacterium pseudotuberculosis* MB154 | target | | NZ_CP024442.1 |
| *Corynebacterium pseudotuberculosis* MB11 | target | | NZ_CP013260.1 |
| *Corynebacterium glutamicum* SCgG1 | target | | NC_021351.1 |
| *Corynebacterium pseudotuberculosis* 12C | target | | NZ_CP011474.1 |
| *Corynebacterium flavescens* OJ8 | target | | NZ_CP009246.1 |
| *Corynebacterium sp.* 2184 | target | | NZ_CP026948.1 |
| *Corynebacterium pseudotuberculosis* OVIAF1 | target | | NZ_CP034410.1 |
| *Corynebacterium ureicelerivorans* IMMIB RIV-2301 | target | | NZ_CP009215.1 |
| *Corynebacterium pseudotuberculosis* T1 | target | | NZ_CP015100.1 |
| *Corynebacterium pseudotuberculosis* FRC41 | target | x | NC_014329.1 |
| *Corynebacterium cystitidis* NCTC11863 | target | | NZ_LT906473.1 |
| *Corynebacterium deserti* GIMN1.010 | target | | NZ_CP009220.1 |
| *Corynebacterium ulcerans* PO100/5 | target | | NZ_CP021417.1 |
| *Corynebacterium pseudotuberculosis* 267 | target | | NC_017462.1 |
| *Corynebacterium humireducens* DSM 45392 | target | | NZ_CP005286.1 |
| *Corynebacterium choanis* 200CH | target | | NZ_CP033896.1 |
| *Corynebacterium maris* DSM 45190 | target | | NC_021915.1 |
| *Corynebacterium pseudotuberculosis* 35 | target | | NZ_CP015185.1 |

| | | | |
|---|---|---|---|
| *Corynebacterium imitans* NCTC13015 | target | | NZ_LT906467.1 |
| *Corynebacterium pseudotuberculosis* CR07 | target | | NZ_CP035715.1 |
| *Corynebacterium halotolerans* YIM 70093 = DSM 44683 | target | | NC_020302.1 |
| [Brevibacterium] flavum ATCC 15168 | target | | NZ_CP011309.1 |
| *Corynebacterium glutamicum* XV | target | | NZ_CP018175.1 |
| *Corynebacterium ulcerans* 210932 | target | | NZ_CP009500.1 |
| *Corynebacterium diphtheriae* NCTC7838 | target | | NZ_LR134537.1 |
| *Corynebacterium glutamicum* CP | target | | NZ_CP012194.1 |
| *Corynebacterium minutissimum* NCTC10288 | target | | NZ_LS483460.1 |
| *Corynebacterium pseudotuberculosis* MB30 | target | | NZ_CP013262.2 |
| *Corynebacterium atypicum* R2070 | target | | NZ_CP008944.1 |
| *Corynebacterium aquilae* S-613 | target | | NZ_CP009245.1 |
| *Corynebacterium ulcerans* 210931 | target | | NZ_CP009583.1 |
| *Corynebacterium ulcerans* NCTC8639 | target | | NZ_LS483416.1 |
| *Corynebacterium variabile* DSM 44702 | target | | NC_015859.1 |
| *Corynebacterium diphtheriae* INCA 402 | target | | NC_016783.1 |
| *Corynebacterium pseudotuberculosis* OVI03 | target | | NZ_CP026524.1 |
| *Corynebacterium glutamicum* K051 | target | | NC_020519.1 |
| *Corynebacterium pseudotuberculosis* 99MAT | target | | NZ_CP036169.1 |
| *Corynebacterium pseudotuberculosis* NCTC4656 | target | | NZ_LR590478.1 |
| *Corynebacterium pseudotuberculosis* 31 | target | | NC_017730.3 |
| *Corynebacterium pseudotuberculosis* 1/06-A | target | | NC_017308.1 |
| *Corynebacterium doosanense* CAU 212 | target | | NZ_CP006764.1 |
| *Corynebacterium aurimucosum* DSM 44827 ATCC 700975 | target | x | NC_012590.1 |
| *Corynebacterium pseudotuberculosis* C231 | target | x | NC_017301.1 |
| *Corynebacterium ulcerans* NCTC10285 | target | | NZ_LR134339.1 |
| *Corynebacterium glutamicum* ZL-6 | target | | NZ_CP004062.1 |
| *Corynebacterium glaucum* DSM 30827 | target | | NZ_CP019688.1 |
| *Corynebacterium jeikeium* FDAARGOS_328 | target | | NZ_CP022054.2 |
| *Corynebacterium pseudotuberculosis* SigmaE | target | | NZ_CP020356.1 |
| *Corynebacterium pseudotuberculosis* KM01 | target | | NZ_CP024995.1 |
| *Corynebacterium jeikeium* NCTC11914 | target | | NZ_LS483459.1 |
| *Corynebacterium renale* NCTC7448 | target | | NZ_LS483464.1 |
| *Corynebacterium ammoniagenes* DSM 20306 = 9.6 | target | | NZ_CP009244.1 |
| *Corynebacterium pseudotuberculosis* 33 | target | | NZ_CP015184.1 |
| *Corynebacterium diphtheriae* 241 | target | | NC_016782.1 |
| *Corynebacterium jeikeium* K411 = NCTC 11915 | target | x | NC_007164.1 |
| *Corynebacterium pseudotuberculosis* MB14 | target | | NZ_CP013261.1 |
| *Corynebacterium pseudotuberculosis* 262 | target | | NZ_CP012022.2 |
| *Corynebacterium pseudotuberculosis* OVI2C | target | | NZ_CP026374.1 |
| *Corynebacterium pseudotuberculosis* Cap1C | target | | NZ_CP038431.1 |
| *Corynebacterium glutamicum* HA | target | | NZ_CP025534.1 |
| *Corynebacterium pseudotuberculosis* 226 | target | | NZ_CP010889.1 |

| | | |
|---|---|---|
| *Corynebacterium pseudotuberculosis* MEX1 | target | NZ_CP017711.1 |
| *Corynebacterium diphtheriae* HC01 | target | NC_016786.1 |
| *Corynebacterium pseudotuberculosis* VD57 | target | NZ_CP009927.1 |
| *Corynebacterium pseudotuberculosis* phoP | target | NZ_CP019768.1 |
| *Corynebacterium pseudotuberculosis* E55 | target | NZ_CP014341.1 |
| *Corynebacterium pseudotuberculosis* PA02 | target | NZ_CP015309.1 |
| *Corynebacterium pseudotuberculosis* 3/99-5 | target | NC_016781.1 |
| *Corynebacterium pseudotuberculosis* 87MAT | target | NZ_CP035719.1 |
| *Corynebacterium testudinoris* DSM 44614 | target | NZ_CP011545.1 |
| *Corynebacterium marinum* DSM 44953 | target | NZ_CP007790.1 |
| *Corynebacterium pseudotuberculosis* MEX29 | target | NZ_CP016826.1 |
| *Corynebacterium pseudotuberculosis* OVI1FL | target | NZ_CP039633.1 |
| *Corynebacterium pseudotuberculosis* PA04 | target | NZ_CP019587.1 |
| *Corynebacterium simulans* Wattiau | target | NZ_CP014635.1 |
| *Corynebacterium camporealensis* DSM 44610 | target | NZ_CP011311.1 |
| *Corynebacterium pseudotuberculosis* CS_10 | target | NZ_CP008923.1 |
| *Corynebacterium pseudotuberculosis* PA01 | target | NZ_CP013327.1 |
| *Corynebacterium ulcerans* FRC58 | target | NZ_CP011913.1 |
| *Corynebacterium pseudotuberculosis* MEX30 | target | NZ_CP017291.1 |
| *Corynebacterium urealyticum* NCTC12011 | target | NZ_LT906481.1 |
| *Corynebacterium phocae* M408/89/1 | target | NZ_CP009249.1 |
| *Corynebacterium diphtheriae* BQ11 | target | NZ_CP029644.1 |
| *Corynebacterium geronticis* W8 | target | NZ_CP033897.1 |
| *Corynebacterium pseudotuberculosis* CIP 52.97 | target | NC_017307.2 |
| *Corynebacterium pseudopelargi* 812CH | target | NZ_CP033898.1 |
| *Corynebacterium pseudotuberculosis* MB20 | target | NZ_CP016829.1 |
| *Corynebacterium striatum* 215 | target | NZ_CP024931.1 |
| *Corynebacterium glutamicum* SCgG2 | target | NC_021352.1 |
| *Corynebacterium pseudotuberculosis* Cap5W | target | NZ_CP039866.1 |
| *Corynebacterium diphtheriae* 31A | target | NC_016799.1 |
| *Corynebacterium vitaeruminis* DSM 20294 | target | NZ_CP004353.1 |
| *Corynebacterium diphtheriae* CDCE 8392 | target | NC_016785.1 |
| *Corynebacterium diphtheriae* NCTC11397 | target | NZ_LN831026.1 |
| *Corynebacterium glyciniphilum* AJ 3170 | target | NZ_CP006842.1 |
| *Corynebacterium diphtheriae* HC04 | target | NC_016788.1 |
| *Corynebacterium pseudotuberculosis* I19 | target | NC_017303.2 |
| *Corynebacterium diphtheriae* C7 (beta) | target | NC_016801.1 |
| *Corynebacterium provencense* 17KM38 | target | NZ_CP024988.1 |
| *Corynebacterium pseudotuberculosis* MEX9 | target | NZ_CP014543.1 |
| *Corynebacterium ulcerans* BR-AD22 | target | NC_015683.1 |
| *Corynebacterium uterequi* DSM 45634 | target | NZ_CP011546.1 |
| *Corynebacterium pseudotuberculosis* P54B96 | target | NC_017031.1 |
| *Corynebacterium ulcerans* 809 | target | NC_017317.1 |

| | | |
|---|---|---|
| *Corynebacterium ulcerans* 05146 | target | NZ_CP009716.1 |
| *Corynebacterium pseudotuberculosis* CAPJ4 | target | NZ_CP026499.1 |
| *Corynebacterium terpenotabidum* Y-11 | target | NC_021663.1 |
| *Corynebacterium simulans* PES1 | target | NZ_CP014634.1 |
| *Corynebacterium matruchotii* NCTC10206 | target | NZ_LR134504.1 |
| *Corynebacterium pseudotuberculosis* E7 | target | NZ_CP036535.1 |
| *Corynebacterium mustelae* DSM 45274 | target | NZ_CP011542.1 |
| *Corynebacterium frankenforstense* ST18 | target | NZ_CP009247.1 |
| *Corynebacterium jeikeium* FDAARGOS_574 | target | NZ_CP033784.1 |
| *Corynebacterium kutscheri* DSM 20755 | target | NZ_CP011312.1 |
| *Corynebacterium xerosis* GS 1 | target | NZ_CP032788.1 |
| *Corynebacterium pseudotuberculosis* MB295 | target | NZ_CP026501.1 |
| *Corynebacterium pelargi* 136/3 | target | NZ_CP035299.1 |
| *Corynebacterium pseudotuberculosis* MB302 | target | NZ_CP021982.1 |
| *Corynebacterium pseudotuberculosis* 29156 | target | NZ_CP010795.1 |
| *Corynebacterium diphtheriae B-D-16-78* | target | NZ_CP018331.1 |
